# Sociodemographic Predictors of Survival in Differentiated Thyroid Cancer: Results from the SEER Database

**DOI:** 10.5402/2012/384707

**Published:** 2012-08-16

**Authors:** Lily E. Johnston, Hop S. Tran Cao, David C. Chang, Michael Bouvet

**Affiliations:** ^1^Department of Surgery, University of California San Diego, San Diego, CA 92103, USA; ^2^Department of Surgery, Moores UCSD Cancer Center, 3855 Health Science Drive 0987, La Jolla, CA 92093-0987, USA

## Abstract

*Background*. Differentiated thyroid carcinoma (DTC) is prognosticated upon a combination of tumor characteristics, such as histology and stage, and patient age. DTC is also notable for having a strong female predominance. Using a nationwide database with long follow-up times, we explored the interplay between tumor biology and patient characteristics in predicting mortality. *Methods*. The Surveillance, Epidemiology, and End Results (SEER) registry data 1973–2005 was examined for patients with DTC as their only known malignancy. Cox multivariate analyses were used to generate mortality hazard ratios to evaluate the effects of age, gender, ethnicity, and marital status. *Results*. We identified 55,995 patients with DTC as their only malignancy. Consistent with the existing literature, the tumors are primarily diagnosed in women (77.5%), and predominantly affect Caucasians (78.3%). Female gender had a protective effect resulting in a 37% decrease in mortality. Age at diagnosis predicted mortality over age 40. Black ethnicity was associated with a 51% increase in mortality compared to Caucasians. *Conclusion*. Multiple demographic factors predict mortality in patients with DTC after adjusting for tumor characteristics, and they appear to have complex interactions. Recognizing the importance of these factors may enable clinicians to better tailor therapy.

## 1. Background

Papillary thyroid carcinoma (PTC) and follicular thyroid carcinoma (FTC) together comprise differentiated thyroid cancers (DTCs), a group of relatively indolent tumors with generally favorable prognosis and good long-term survival. DTC is also relatively uncommon, accounting for approximately 1-2% of all cancers in the United States, and up to 5% of cancers worldwide [1]. However, the incidence of thyroid cancer is increasing in the United States, a trend almost entirely attributed to an increase in PTC [2–5]. Numerous studies have examined both tumor and patient factors that influence prognosis in DTC, but the indolent nature of the disease is a challenge for researchers since unusually long patient follow-up times are required for optimal data analysis. Even 10-year survival data may be insufficient for identifying subtle prognostic findings in the context of tumors that can recur decades after initial presentation [6].

The goal of this study was to review tumor histology and patient sociodemographic factors that may affect prognosis and to reexamine these findings and their interactions in a large retrospective database with long follow-up times. The results may help clinicians identify higher-risk patient populations that could benefit from targeted interventions such as more intense followup or increased social support following definitive treatment, irrespective of their tumor burden. 

## 2. Methods 

### 2.1. SEER Registry and Study Population

 The Surveillance, Epidemiology, and End Results (SEER) project is a United States, population-based cancer registry started in 1973 and is supported by the National Cancer Institute and Centers for Disease Control and Prevention. SEER contains data on cancer incidence, prevalence, mortality, and population-based variables, representing now approximately 28 percent of the United States population sampled across multiple geographic regions. The SEER data set also contains information on the primary characteristics of the tumor, including site, spread, and histology when available, as well as limited information regarding treatment excluding chemotherapy.

### 2.2. Data Collection and Analysis

 This study was reviewed and approved by the Institutional Review Board at the University of California, San Diego. We examined SEER data between 1973 and 2005 and selected patients with a diagnosis of well-differentiated thyroid carcinoma as their only known malignancy, as defined by a combination of International Classification of Diseases for Oncology (ICD-O) site code of C73.9 (i.e., thyroid), papillary or follicular histology consistent with World Health Organization categories, and tumor sequence number equal to zero. Patients with less than one month of followup were excluded from the study.

Tumor stage was defined using current AJCC TNM staging criteria for differentiated thyroid cancer. Due to coding overlap from older data, tumor categories T4a and T4b could not be discerned, so that stages IVa and IVb were combined as stage IVa/b. Categories of race/ethnicity as defined in SEER were Caucasian, Black, Asian, Hispanic, American Indian/Alaskan Native, and other/unknown. Marital status was defined as married, never married, divorced, separated or widowed; the last three categories were combined in our analyses as “previously married.” Patients were also separated by their SEER geographic registry to explore possible geographic differences in DTC outcomes; unless otherwise stated, geographic hazard ratios are standardized against outcomes in the San Francisco/Oakland registry, which has been collecting data since 1973. In univariate analyses, log-rank tests were used to compare survival functions, and Kaplan-Meier curves used to display these functions. We used Cox multivariate proportional hazard models to generate relative risk of death by any cause with 95% confidence intervals, controlling for stage, histology, surgical and radiation treatment. Subset analyses explored the influence and interaction of other variables, including gender, ethnicity, marital status, stage at diagnosis, age at diagnosis, and treatment modality. Age at diagnosis was categorized into patients diagnosed at 30 years of age or younger, and then increasing in 10-year intervals beginning at age 31. 

### 2.3. Statistics

 Analyses were performed using the STATA 10 (Stata Corp., College Station, TX, USA) software package. Statistical significance was defined as a Type I error probability of <0.05; all confidence intervals (CI) are reported as 95% CI.

## 3. Results

### 3.1. General Findings

 We identified 55,995 patients with papillary or follicular thyroid disease as their only known malignancy. Demographic characteristics of this sample are noted in Table 1. Of these, 49,796 had complete data to generate a TNM stage based upon the AJCC Cancer Staging Manual, 6th edition. Consistent with the existing body of literature, the majority of tumors are diagnosed in women (77.5%), and DTC predominantly affects Caucasians (78.3%). Age at diagnosis ranged from 2–100 years of age, with a mean of 44.8 (SD: 15.6) and a median of 43 years of age. The majority of patients are diagnosed in stage I (76.5%), though the mortality difference between stage I and stage II disease in the multivariate analysis just failed to meet statistical significance (stage II HR: 1.24, 95% CI: 1.0–1.52). Approximately 99% of those patients who had data regarding surgical treatment did have surgery (*n* = 39,056). Less than half of patients recorded had radioactive iodine treatment (*n* = 21,444; 42.2%). The majority of patients were married at the time of diagnosis (*n* = 35, 918; 66.13%).

In our multivariate analysis, protective demographic factors include female gender (HR: 0.63, 95% CI: 0.57–0.71) and being married versus never married (HR: 0.69, 95% CI: 0.59–0.8). Of the non-Caucasian ethnic groups, only Blacks had a statistically significant increased mortality risk when compared with non-Hispanic Caucasians (HR: 1.45, 95% CI: 1.2–1.77). 

### 3.2. Papillary versus Follicular Carcinoma

Papillary cancers account for 87.5 percent of DTC (*n* = 49,012). Patients with PTC were typically younger at diagnosis (mean 44.2 years versus 49.2, *P* < 0.0001), and fewer were diagnosed with advanced stage disease (16.0% stage 3 or 4 versus 25.4% in FTC). Tumor size and distant metastases, rather than nodal spread, appear to account for the increased stage of FTC patients at diagnosis (Table 2). Men account for a slightly higher proportion of FTC patients than PTC patients (26.6% versus 21.9%, resp.). PTC and FTC patients were equally likely to have surgery (99.1% versus 98.9%), but PTC patients were slightly more likely to receive radioiodine therapy (42.7% versus 38.7%). Follicular histology accounts for 21.3% of DTC in the Black population, a significantly higher proportion than for any other racial or ethnic group (all others <15%).

 In multivariate analyses, follicular histology (versus papillary) is associated with increased mortality (HR: 1.19, 95% CI: 1.04–1.36). Unlike PTC, where there is no difference in mortality risk between stage I and stage II disease (HR: 1.12, 95% CI: 0.88–1.45), stage II disease nearly doubles the mortality risk in FTC (HR: 1.98, 95% CI: 1.21–3.25).

### 3.3. Patient Age Effects

Replicating a well-documented finding for DTC, increasing age is significantly correlated with increased mortality in our multivariate analysis. Compared with patients under the age of 30, there is a steep, steady, and significant rise in mortality risk as age increases in 10-year increments. Compared to patients under 30, patients ages 30–40 have an approximately 65% increase in mortality risk (HR: 1.66, 95% CI: 1.28–2.13); the hazard ratios roughly double for every 10-year increase until patients are 90 and older. Kaplan-Meier survival functions were calculated for these age groups and they were found to be statistically different using the log-rank method (*P* < 0.0001).

 Subset analyses examined outcomes by decade of life after age 30. In patients 30 and under, treatment with radioactive iodine but not surgery predicts mortality, whereas patients 31–40 appear to benefit from surgery but not radioactive iodine. After age 50, mortality outcomes no longer appear to be significantly influenced by race/ethnicity, marital status, or histology, illustrating the significance of advanced age in DTC outcomes.

### 3.4. Patient Gender Effects

There exist a number of critical differences in the presentation of DTC in men and women that may clarify the protective effect of female gender. First, less than 15% of women presented with stage 3 or greater disease at the time of diagnosis, compared to more than 25% of men. Men were slightly more likely to have follicular cancer than women (14.8% versus 11.8%). Men were also on average over three years older than women at the time of diagnosis (47.6 years versus 44.0, *P* < 0.0001); this will in part account for the higher stage at time of diagnosis based on age-centered AJCC guidelines. Still, when adjusting for stage, age, and histology, men fared worse than women (HR: 1.58, 95% CI: 1.41–1.77). The effect of increasing age is significant for both women and men, but that the magnitude of that effect appears more pronounced in women (see Table 3). Additionally it appears that increased mortality related to age begins early in both women and men: women 31–40 years old had a 69% increase in mortality hazard compared to their counterparts 30 and younger (HR: 1.69, 95% CI: 1.25–2.30); men 31–40 similarly show a 62% increase in mortality hazard compared to men 30 and under (HR: 1.62, 95% CI: 1.01–2.59).

**Figure 1:**
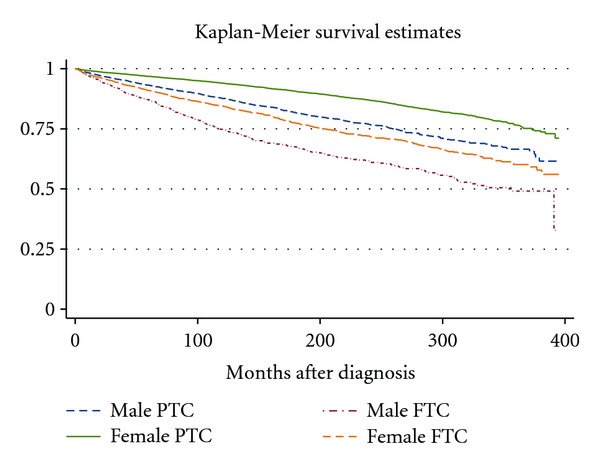
Kaplan-Meier survival curves illustrate how overall mortality changes with gender and histology. Although overall survival with DTC is good, especially at 5 and 10 years, both follicular histology and male gender continue to be risk factors for poor prognosis as far as 30 years after diagnosis. Log-rank test *P* < 0.0001.

Univariate analysis finds significant differences when looking at gender and histology interactions (Figure 1). In the Cox regression, follicular cancer was associated with significantly poorer prognosis than papillary cancer in men, but not women (HR: 1.37, 95% CI 1.10–1.71). 

Women in specific geographic regions had poorer prognosis relative to their counterparts in San Francisco than did men, including metropolitan Detroit (HR: 1.36, 95% CI 1.03–1.8), Los Angeles (HR: 1.43, 95% CI 1.09–1.88), and Kentucky (HR: 2.43, 95% CI 1.31–4.5). Treatment effects also differed between genders: surgery was highly protective in both men and women, whereas radioactive iodine is protective in women but not men (Table 3).

### 3.5. Patient Ethnicity Effects

**Figure 2:**
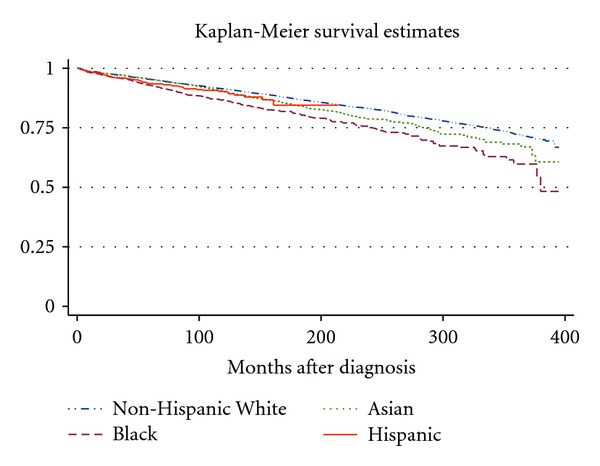
Kaplan-Meier survival curves illustrating the long-term overall mortality outcomes based upon race and ethnic group. Log-rank test *P* < 0.0001.

Non-Hispanic Caucasians were more likely to be diagnosed with stage I disease (77.6%) compared to their Black, Asian, and Hispanic counterparts (72.4, 72.8, and 70.3%, resp.). All ethnic groups were more likely to present with stage IV disease than non-Hispanic Caucasians (Table 4). As discussed above, non-Hispanic Caucasians are most likely to be diagnosed with DTC, whereas Blacks have the highest mortality risk.  Figure 2 illustrates that non-Hispanic White patients have the best survival followed by Asian then Black patients. Follow-up times for Hispanic patients are more limited, but outcomes appear to trend with those of Asian patients. Beyond these general effects, race/ethnicity appears to have significant interactions with other risk factors discussed above. For example, increasing age does not affect outcomes in Black or Asian patients until 51–60 years old (Table 5). Another finding was that neither treatment with radioactive iodine (HR: 0.75, 95% CI: 0.38–1.46) nor surgery (HR: 0.32, 95% CI: 0.06–1.60) predicted decreased mortality in Hispanic patients. There were also interactions between race/ethnicity and geographic location as described below.

### 3.6. Geographic Effects

In the multivariate analysis, three registry areas had increased mortality risk compared to patients in the San Francisco registry: metropolitan Detroit, Hawaii, and Los Angeles. Significant interactions between race/ethnicity and geography were also noted: the only geographic area with significantly different mortality risk for non-Hispanic Caucasian patients is metropolitan Detroit (HR: 1.29, 95% CI: 1.01–1.66). In contrast, Black patients have poorer outcomes in New Mexico, Seattle/Puget Sound, and greater California (excluding Los Angeles, San Jose, and the San Francisco bay area). Asian patients also fared worse in Hawaii.

Treatment effects were also inconsistent between regions: surgery only reduces mortality risk in certain registry regions (Table 6).

## 4. Discussion

Multiple retrospective case series and registry studies have examined important prognostic factors in DTC, but many of these studies are dated and are also limited by follow-up times that may be insufficient to detect subtle effects that may be related to mortality in patients with this indolent malignancy [6–19]. Our study sought to reevaluate the role of histology and patient factors in DTC outcomes by analyzing the Surveillance, Epidemiology, and End Results (SEER) registry 1973–2005, which offers the advantages of covering a large and diverse population and avoiding potential selection, referral, and other biases inherent to single institution studies. Even though SEER data date back to 1973, many studies evaluate prognosis in terms of 5- and 10-year survival and do not take advantage of follow-up times that may in fact be significantly longer than this. By using Cox multivariate analyses and expressing risk in terms of overall hazard ratio, we take maximal advantage of the available data. However, databases like the SEER registry have inherent limitations that must, on one hand, be taken into consideration when interpreting our results. For example, we were unable to distinguish between stages IVa and IVb due to coding overlaps across multiple time periods. Furthermore, information such as family history, vascular invasion, or other histologic findings was not evaluated nor included in our dataset. Treatment, which influences survival, was grossly controlled but does not include reliable data on extent of surgery, radioactive iodine protocols, and other treatment variables that may have a high degree of variability across time and locations. Finally, although there are some data on cause of death in SEER, these come from death certificates and thus are not independently verified and may be highly inaccurate especially when looking at a generally indolent malignancy; thus, we have used all-cause mortality as the endpoint in this study. On the other hand, large population-based registries offer a more accurate reflection of actual practice and outcomes than individual institutional studies and are a valuable resource for outcome-directed research.

## 5. Age

Age at diagnosis is the most well-known patient variable predicting prognosis, so much so that it is an integral part of almost every staging protocol and risk model for DTC [6, 8, 10, 12, 13, 17, 19, 20]. In the early literature, there was thought to be a stepwise increase in mortality once patients were above a certain age. However, the precise age at which this step function took place was not entirely clear [6, 8, 12, 13]. Our data demonstrate a steady increase in all-cause mortality risk as a function of age, though this in part reflects the inherent increase in mortality hazard with increasing age even in healthy individuals. We have demonstrated that in all comers with DTC, there does not appear to be a definitive age group in which there is a sudden increase in mortality, and rather that mortality increases steadily with age as it does with many other disease processes. However, subset analyses reveal that this may not be universally true for all patient populations: women with DTC appear to be much more sensitive to the effects of increasing age, and Black and Asian patients may in fact have a stepwise increase in mortality as a function of age, but this appears to occur somewhat later (age 51–60) than current staging guidelines suggest.

## 6. Gender

Many authors describe poorer outcomes in men with DTC than women [8, 12, 18, 21], though others find no effect of gender on mortality [7, 17]; thus, there is weak consensus regarding the effect of gender on outcomes of DTC. Our data strongly indicate that female gender is associated with significantly reduced mortality risk, which is more pronounced in certain subpopulations. The discussion as to why DTC affects women versus men by a 3 to 1 ratio is beyond the scope of this paper, but others have examined hormonal and other mechanisms; it could be that these same mechanisms also account for the 37% mortality risk reduction in women, and perhaps why treatment with radioactive iodine reduces mortality risk in women but not in men [22].

## 7. Ethnicity and Geography

In the multivariate analyses, our findings are consistent with others previously reported, showing that only Black ethnicity is associated with increased mortality hazard ratios [9]. However, the subgroup analyses reflect a more nuanced picture with histology, geographic location, and treatment all interacting with a patient's race/ethnicity. Morris et al. have found evidence that suggests blacks may have both inferior screening compared to white counterparts as well as slightly less aggressive disease [23]; Brown and colleagues find no survival difference between Blacks and Whites in a military system where all patients have equal access to funded healthcare [24]. Other studies have examined changing incidence across geographic areas, but have not examined the interaction effects of geography with race/ethnicity [23]. Although our data do not speak to the underlying cause of the disparity between regions and ethnic groups within those regions, they do suggest that a region-specific analysis may be warranted when evaluating disparities in DTC outcomes.

## 8. Conclusion

Factors known to influence survival in DTC include histology, stage, patient age, gender, and race/ethnicity. Examining these factors using a large dataset with long follow-up times permits a more nuanced understanding of their impact and interactions. This study also reveals that even within the United States, geography and race/ethnicity have unique interactions that appear to impact outcomes. Further research is needed to corroborate these apparent disparities and suggest possible mechanisms. Recognizing the subtle but meaningful way these variables impact mortality will allow clinicians to better tailor treatment and followup to particularly high-risk patient populations.

## Figures and Tables

**Table 1:** General patient characteristics.

	Total *N* (%)	Papillary *N* (%)	Follicular *N* (%)
Gender			
Male	12,580 (22.5)	10,722 (21.9)	1,858 (26.6)
Female	43,415 (77.5)	38,290 (78.1)	5,125 (73.4)

Age at diagnosis			
<30	9,489 (17.0)	8,522 (17.4)	967 (13.9)
31–40	13,143 (23.5)	11,843 (24.2)	1,300 (18.6)
41–50	13,539 (24.2)	12,071 (24.6)	1,468 (21.0)
51–60	9,765 (17.4)	8,526 (17.4)	1,239 (17.7)
61–70	5,575 (10.0)	4,672 (9.5)	903 (12.9)
71–80	3,350 (6.0)	2,560 (5.2)	790 (11.3)
81–90	1,048 (1.9)	758 (1.6)	290 (4.2)
>90	86 (0.15)	60 (0.1)	26 (0.4)

Ethnicity			
Non-Hispanic White	39,530 (78.3)	34,586 (78.5)	4,944 (77.0)
Black	2,990 (5.9)	2,354 (5.3)	636 (9.9)
Asian	5,883 (11.7)	5,225 (11.9)	658 (10.3)
Hispanic	1,559 (3.1)	1,432 (3.3)	127 (2.0)
Native American/Alaskan	330 (0.65)	299 (0.7)	31 (0.5)
Unknown/other	169 (0.33)	146 (0.3)	23 (0.4)

Treatment			
Surgery	39,056 (99.0)	33,901 (99.1)	5,155 (98.6)
Radioactive iodine	21,444 (42.2)	25,607 (57.3)	3,724 (61.3)

**Table 2:** Tumor staging characteristics.

Tumor presentation	Total *N* (%)	Papillary *N* (%)	Follicular *N* (%)
Stage			
I	38,080 (76.5)	34,663 (78.5)	3,417 (60.6)
II	3,224 (6.47)	2,431 (5.51)	793 (14.1)
III	5,361 (10.8)	4,387 (9.94)	974 (17.3)
IVa/IVb	2,358 (4.74)	2,176 (4.93)	182 (3.23)
IVc	773 (1.55)	496 (1.12)	277 (4.91)

T			
1	22,723 (49.4)	21,618 (52.8)	1,105 (21.9)
2	9,650 (21.0)	7,842 (19.1)	1,808 (35.8)
3	11,654 (25.3)	9,718 (23.7)	1,936 (38.4)
4	1,988 (4.32)	1,793 (4.38)	195 (3.87)

N			
0	31,052 (76.1)	26,999 (73.9)	4,053 (95.0)
1a	5,517 (13.5)	5,404 (14.8)	113 (2.65)
1b	4,215 (10.3)	4,116 (11.3)	99 (2.32)

M			
0	54,958 (98.2)	48,277 (98.5)	6,681 (95.7)
1	1,037 (1.9)	735 (1.5)	302 (4.32)

*Percentages may not add up to 100 due to rounding.

**Table 3:** Gender effects.

	Unadjusted HR (95% CI)	Women (95% CI)	Men (95% CI)
Age at diagnosis			
<30	1.0 (reference)	1.0 (reference)	1.0 (reference)
31–40	1.66 (1.29–2.15)	1.69 (1.24–2.30)	1.62 (1.01–2.59)
41–50	3.17 (2.46–4.10)	2.89 (2.12–3.94)	3.57 (2.24–5.7)
51–60	5.55 (4.17–7.39)	5.84 (4.14–8.25)	4.76 (2.81–8.06)
61–70	13.7 (10.4–18.1)	15.6 (11.2–21.7)	10.8 (6.44–18.2)
71–80	29.3 (22.2–38.6)	33.8 (24.4–47.0)	22.9 (13.6–38.7)
81–90	61.5 (45.5–83.1)	80.3 (56.2–114.6)	35.2 (19.6–63.1)
>90	99.6 (57.9–171)	124.1 (65.1–236.3)	51.4 (17.9–147.8)

Histology			
Papillary	1.0 (reference)	1.0 (reference)	1.0 (reference)
Follicular	1.19 (1.04–1.36)	1.08 (0.91–1.27)	1.37 (1.1–1.71)

Treatment			
Radioactive Iodine	0.85 (0.76–0.96)	0.78 (0.68–0.91)	1.0 (0.82–1.22)
Surgery	0.42 (0.31–0.56)	0.57 (0.4–0.83)	0.24 (0.15–0.41)

**Table 4:** Stage and ethnicity.

Stage at diagnosis	Non-Hispanic White *N* (%)	Black *N* (%)	Asian *N* (%)	Hispanic *N* (%)
I	27,035 (77.6)	1,914 (72.4)	3,799 (72.8)	1,046 (70.3)
II	2,252 (6.47)	219 (8.29)	308 (5.90)	109 (7.33)
III	3,572 (10.3)	352 (13.3)	634 (12.1)	193 (13.0)
IVa/IVb	1,530 (4.39)	84 (3.18)	341 (6.53)	98 (6.59)
IVc	442 (1.27)	74 (2.80)	139 (2.66)	42 (2.82)

**Table 5:** Race/ethnicity effects.

	Non-Hispanic Caucasian HR (95% CI)	Black HR (95% CI)	Asian HR (95% CI)	Hispanic HR (95% CI)
Age at diagnosis				
<30	1.0 (reference)	1.0 (reference)	1.0 (reference)	1.0 (reference)
31–40	1.81 (1.34–2.44)	1.06 (0.49–2.29)	1.29 (0.63–2.64)	1.90 (0.19–18.7)
41–50	3.58 (2.66–4.82)	1.98 (0.91–4.34)	1.93 (0.93–3.99)	4.96 (0.55–44.8)
51–60	6.14 (4.38–8.59)	4.01 (1.65–9.72)	3.55 (1.59–7.91)	7.46 (0.78–71.5)
61–70	16.1 (11.7–22.3)	7.72 (3.15–18.9)	8.58 (3.90–18.9)	11.7 (1.20–115)
71–80	39.9 (28.9–55.0)	12.8 (4.96–33.0)	11.8 (5.28–26.2)	28.1 (3.01–262)
81–90	78.4 (55.2–111)	30.3 (10.6–87.2)	25.8 (10.6–62.5)	69.5 (7.01–689)
>90	160 (88.2–290)	—	25.6 (4.69–139)	—

Histology				
Papillary	1.0 (reference)	1.0 (reference)	1.0 (reference)	1.0 (reference)
Follicular	1.16 (0.99–1.35)	1.33 (0.89–1.99)	1.36 (0.93–1.99)	0.81 (0.32–2.06)

Treatment				
Radioactive iodine	0.93 (0.81–1.07)	0.90 (0.59–1.38)	0.58 (0.42–0.80)	0.75 (0.38–1.46)
Surgery	0.42 (0.29–0.60)	0.30 (0.11–0.83)	0.48 (0.21–1.07)	0.32 (0.06–1.60)

SEER registry location				
San Francisco/Oakland	1.0 (reference)	1.0 (reference)	1.0 (reference)	1.0 (reference)
Connecticut	1.03 (0.79–1.34)	0.86 (0.31–2.39)	0.42 (0.09–1.83)	
Metropolitan Detroit	1.29 (1.01–1.66)	1.68 (0.85–3.34)	0.89 (0.12–6.58)	
Hawaii	1.51 (0.94–2.45)	3.89 (0.82–18.5)	1.79 (1.21–2.66)	
Iowa	1.09 (0.84–1.41)	—	1.05 (0.24–4.50)	
New Mexico	1.0 (0.71–1.42)	6.20 (1.29–29.8)	1.76 (0.24–13.0)	2.46 (0.41–14.9)
Seattle (Puget Sound)	1.1 (0.84–1.44)	3.75 (1.32–10.7)	1.46 (0.70–3.05)	—
Utah	1.08 (0.79–1.48)	—	—	—
Metropolitan Atlanta	1.17 (0.86–1.60)	1.17 (0.53–2.61)	0.54 (0.12–2.44)	10.9 (0.85–141)
Alaska		—	—	—
San Jose/Monterey	0.86 (0.54–1.35)	2.50 (0.31–20.3)	1.68 (0.86–3.29)	0.89 (0.15–5.13)
Los Angeles	1.17 (0.90–1.53)	2.01 (0.95–4.26)	1.42 (0.90–2.24)	1.49 (0.41–5.36)
Rural Georgia	1.56 (0.38–6.34)	—	—	—
Greater California (excluding above regions)	1.02 (0.73–1.44)	2.82 (1.04–7.66)	1.04 (0.43–2.51)	1.94 (0.44–8.60)
Kentucky	1.68 (0.95–2.96)	—	—	—
Louisiana	1.1 (0.66–1.83)	2.23 (0.87–5.71)	—	—
New Jersey	0.93 (0.64–1.35)	0.22 (0.03–1.77)	2.54 (0.96–6.75)	—

**Table 6:** Treatment effects by location.

SEER registry location	Surgery HR (95% CI)	Radioactive iodine
San Francisco/Oakland	0.56 (0.22–1.38)	0.90 (0.60–1.35)
Connecticut	0.50 (0.19–1.30)	1.02 (0.65–1.59)
Metropolitan Detroit	0.37 (0.08–1.73)	0.91 (0.64–1.30)
Hawaii	0.02 (0.002–0.19)	0.63 (0.40–1.01)
Iowa	0.03 (0.01–0.17)	0.76 (0.51–1.13)
New Mexico	4.15 (0.22–78.8)	1.38 (0.73–2.59)
Seattle (Puget Sound)	0.18 (0.04–0.83)	0.89 (0.59–1.35)
Utah	0.04 (0.01–0.16)	1.22 (0.65–2.28)
Metropolitan Atlanta	0.55 (0.10–3.15)	0.84 (0.50–1.42)
Alaska	—	—
San Jose/Monterey	0.15 (0.01–1.45)	0.89 (0.40–1.95)
Los Angeles	0.56 (0.26–1.22)	0.64 (0.48–0.84)
Rural Georgia	—	—
Greater California (excluding above regions)	0.22 (0.08–0.63)	0.67 (0.39–1.15)
Kentucky	—	0.72 (0.21–2.42)
Louisiana	0.15 (0.04–0.54)	0.73 (0.29–1.81)
New Jersey	0.89 (0.33–2.40)	1.24 (0.63–2.44)
